# Perch Hydrolysates from Upcycling of Perch Side Streams Accelerate Wound Healing by Enhancing Fibroblasts to Secrete Procollagen I, Fibronectin, and Hyaluronan

**DOI:** 10.3390/cimb47010057

**Published:** 2025-01-16

**Authors:** Jia-Feng Chang, Chih-Yu Hsieh, Ling-Ni Chen, Mao-Hsiang Lee, Yi-Han Ting, Chi-Yu Yang, Chih-Cheng Lin

**Affiliations:** 1 Division of Nephrology, Department of Internal Medicine, Taoyuan Branch of Taipei Veterans General Hospital, Taoyuan 330, Taiwan; cjf6699@gmail.com; 2 Department of Nursing, Yuanpei University of Medical Technology, Hsinchu 300, Taiwan; 3 Department of Biotechnology and Pharmaceutical Technology, Yuanpei University of Medical Technology, Hsinchu 300, Taiwan; cyuh0817@gmail.com (C.-Y.H.); celia.chen@topco-global.com (L.-N.C.); sam.lee@topco-global.com (M.-H.L.); serena.ting@topco-global.com (Y.-H.T.); 4 Anyong Biotechnology Inc., Kaohsiung 827, Taiwan; 5 Animal Toxicity Laboratory, Agricultural Technology Research Institute, Hsinchu 300, Taiwan; chiyu@mail.atri.org.tw

**Keywords:** collagen, hydrolysate, perch, short-chain peptides, upcycle, wound healing

## Abstract

Wound healing incurs various challenges, making it an important topic in medicine. Short-chain peptides from fish protein hydrolysates possess wound healing properties that may represent a solution. In this study, perch hydrolysates were produced from perch side steams using a designed commercial complex enzyme via a proprietary pressure extraction technique. The average molecular weight of the perch peptides was 1289 kDa, and 62.60% of the peptides had a low molecular weight (≤1 kDa). Similarly to the beneficial amino acid sequence FPSIVGRP, FPSLVRGP accounted for 6.21% abundance may have a potential antihypertensive effect. The concentrations of collagen composition and branched-chain amino acids were 1183 and 1122 mg/100 g, respectively. In a fibroblast model, active perch peptides accelerated wound healing mainly by increasing the secretion of procollagen I, fibronectin, and hyaluronan. In an SD rat model established to mimic human wounds, orally administered perch hydrolysates with a molecular weight below 2.3 kDa accelerated wound healing, which mainly resulted from collagen-forming amino acids, branched-chain amino acids, and matrikine. Collectively, the residue of perch extract can be upcycled via a hydrolysis technique to produce not only bioactive sequences but also short-chain peptides. Considering the therapeutic potential to promote wound healing, such by-products are of great value and may be developed as dietary nutraceuticals.

## 1. Introduction

Fish soup is a conventional oriental tonic, and fish is an important source of protein for humans. Furthermore, fish soup is ideal for individuals unable to consume fish easily, such as the sick or post-surgery patients. The decomposition and spoilage of fish protein after capture, leading to quick decay, can be attributed to endogenous enzymes and external microbial activity. Extraction technology is extensively used in Taiwan to produce fish essence by extracting the protein from the fish body and sterilizing it. However, after the extraction, a significant amount of side stream and waste is generated, and the fish body cannot be entirely recycled [1]. The aquatic industry considers it essential to improve product value from aquatic side streams and minimize waste [2,3]. Enzymatic hydrolysis is a highly efficient technology to retrieve 80% of protein from the heads of cod (*Gadus morhua*). The extracted protein can be used as an ingredient in dietary supplements intended for human consumption [4].

Peptides typically remain inactive within the parent protein sequence. However, bioactive peptides can be generated through proteolysis using methods like enzymatic hydrolysis or fermentation [5,6]. Numerous food-derived peptides have demonstrated diverse beneficial impacts on human health based on their structure, encompassing antioxidant, anticancer, antihypertensive, antimicrobial, and immunomodulatory effects [7,8,9,10,11]. Studies illustrate that peptides obtained from different types of fish proteins exhibit a range of biological actions [12]. Two marine hydrolysates, extracted from blue whiting (*Micromesistius poutassou*) and brown shrimp (*Penaeus aztecus*), elicited a high level of cholecystokinin secretion in endocrine cells [13]. Furthermore, the peptide originating from the fish protein hydrolysate of croaker (*Otolithes ruber*) was revealed to possess antioxidative properties, increase endurance capacity, and facilitate recovery from oxidative stress in Wistar rats [14]. Consuming fish hydrolysates, consisting primarily of dipeptides and tripeptides, has demonstrated multiple advantages in maintaining skeletal muscle health and metabolism amidst the aging process [15,16]. Enzymatically hydrolyzed peptides extracted from the Pacific oyster (*Crassostrea gigas*), with molecular weights ranging from 550 Da to 2300 Da and peptide chain lengths between 11 and 16 amino acids, have been observed to exhibit anti-inflammatory properties whilst promoting fibroblast proliferation upon topical application [17].

Reducing waste is a worldwide issue for promoting the reduction in carbon emissions within ESG. Collagen peptide and flesh protein peptide extracted from Alaska Pollock showed a significant increase in the wound healing rate in a rat wound model [18]. The process of extracting perch essence, using high temperature and pressure, results in about 66% residue consisting of flesh, bones, head, scales, skin, and fins. Despite numerous investigations into wound healing activity associated with fish byproducts and their hydrolysates [18,19], few studies have analyzed the protein profile of perch hydrolysates as a byproduct of whole perch extraction and their wound healing activity as a dietary supplement for waste reduction. Thus, the purpose of this study is to evaluate the composition characteristics of perch hydrolysate and the effectiveness of its impact on wound healing.

## 2. Materials and Methods

### 2.1. Perch Hydrolysate Preparation

The perch (*Lates calcarifer*) used in this study was supplied by Yilan Anyong Lohas Co., Ltd. (Yilan, Taiwan). The fish were subjected to thorough cleaning procedures that involved removal of the viscera, followed by extraction of the flesh, bone, and scales with RO water (*w*/*v* = 1:1) under high pressure. The resulting extract was filtered to obtain the commercial perch essence. Perch byproduct was mixed with deionized water in a ratio of 1:3 (*w*/*w*). Commercial enzymes, including Protamex, Neutrase, Pepsin, Papain, Trysin, Flavourzyme, and Protana Prime (Novozymes A/S Enzyme Inc., Bagsvaerd, Denmark), were added at a concentration of 0.6% (the enzyme-to-perch-byproduct weight ratio was 0.6:100, *w*/*w*). This was succeeded by hydrolysis under an optimal temperature of enzymes (40~60 °C) (YC-EC-600, Yenchen Machinery Co., Ltd., Taoyuan, Taiwan). The enzymatic reaction was halted at 100 °C for ten minutes. Following this, the resulting hydrolysate was subjected to centrifugation and filtering to obtain perch hydrolysate (PH). The PH underwent filtration using a tangential flow filtration system (Advanced Biotechnology Laboratories Co., Ltd., New Taipei City, Taiwan) with a 30 kDa ultrafiltration membrane (Microdyn-Nadir Ltd., Wiesbaden, Germany) to yield PH30 with MW < 30 kDa. The resulting filtrate was then freeze-dried to produce a powder for experimental analysis.

### 2.2. Determination of Free and Hydrolyzed Amino Acid Compositions

The free and hydrolyzed amino acid compositions were analyzed according to ISO 13903:2005 [20] using an amino acid analyzer (Thermo Fisher Scientific, Inc., Waltham, MA, USA). The perch hydrolysate was extracted with sulphosalicylic acid to precipitate any protein. The resultant sample was brought to volume with a loading buffer and then transferred into a vial. The free amino acid concentration in mg/100 g was calculated by separating amino acids in an amino acid analyzer, followed by detection through post-column derivatization with 440 and 570 nm ninhydrin reagent. Finally, we compared the absorbance intensity of the sample and standard to calculate the free amino acid concentration, and the unit was mg/100 g.

The composition of hydrolyzed amino acids was analyzed in a similar way to that of free amino acids, except that the process of hydrolysis was involved. Drying perch hydrolysates were oxidized with hydrogen peroxide and formic acid at a cold temperature, followed by acid hydrolysis using aqueous hydrochloric acid. The amino acids were separated by ion exchange chromatography and determined by a reaction with ninhydrin, using photometric detection at 570 nm (440 nm for proline) with an amino acid analyzer (Biochrom Ltd., Cambridge, UK).

### 2.3. Analysis of Soluble Protein Content

The soluble protein content was determined using a modified Folin–Lowry method [21]. First, 0.5 mL of diluted sample solution was combined with 0.5 mL reagent A and 4 mL reagent B (Bio-Rad Dc Protein Assay Kit, Bio-Rad Laboratories, Inc., Berkeley, CA, USA). The solution was then shaken and allowed to stand at room temperature for 15 min. Finally, the absorbance was assessed at 540 nm using an SP-UV1100 spectrophotometer from DLAB Scientific Inc. in Beijing, China. Bovine serum albumin was utilized as an analytical standard, and the result was transformed into soluble protein content, measured in mg/mL.

### 2.4. Determination of Peptide Content Based on α-Amino Group Content

Peptide levels were assessed with minor adjustments to the approach employed by [22]. The reaction comprised adding 2 mL of o-phthaldialdehyde (OPA) reagent to 50 µL of the sample solution, which had been diluted. After mixing, the sample solution was allowed to settle for 2 min at room temperature, followed by measuring the absorbance at 340 nm using a spectrophotometer (SP-UV1100, DLAB Scientific Inc., Beijing, China). The α-amino group levels were evaluated using Leu-Gly as a standard (unit: mg/mL).

### 2.5. Determination of Degree of Hydrolysis

The degree of hydrolysis of PH was determined by the OPA method [23]. To fully hydrolyze the perch hydrolysate, 6 N HCl was used in a dry bath at 110 °C for 24 h. The total content of α-amino groups in the perch hydrolysate, fully hydrolyzed perch by-product, and raw material background (the perch by-product was added to deionized water, homogenized for 5 min, then heated in boiling water for 10 min) was determined via the OPA method. A standard calibration curve was prepared with L-leucine standard substance, and the α-amino group content was converted to mg/mL. Furthermore, we utilized the subsequent formula to calculate the degree of hydrolysis.

DH (%) = (α − ni)/(nT − ni)

α: α-amino group content in the perch hydrolysate.
nT: α-amino group content in the fully hydrolyzed perch byproduct.
ni: α-amino group content in the perch byproduct.

### 2.6. Analysis of Protein Profile

The sample solution underwent filtration through a 0.22 µm filter membrane, and 100 µL of the filtrate served as the injection volume. Subsequently, gel filtration chromatography (TSKgel GMPWxL Column, Tosoh Bioscience Inc., South San Francisco, CA, USA) was performed for purification and separation, equilibrated with 0.05 M NaNO_3_. Data analysis was conducted with the Viscotek GPC System (1122 pump, 717 Auto Injector, 270 Dual Detector/Differential Viscometer and Laser Light Scattering Detector, 3580 RI detector, and OmniSEC 4.6 Station, Malvern Panalytical Ltd., Malvern, UK).

### 2.7. Identification of Peptide Sequence by LC-MS/MS Analysis

The peptides were diluted in HPLC buffer A (0.1% formic acid) and loaded onto a reverse-phase column (Zorbax 300SB-C18, 0.3 × 5 mm; Agilent Technologies, Santa Clara, CA, USA ). The desalted peptides were then separated on a homemade column (Waters BEH 1.7 μm, 100 μm I.D. × 10 cm with a 15 μm tip) using a multi-step gradient of HPLC buffer B (99.9% acetonitrile/0.1% formic acid) for 70 min with a flow rate of 0.3 μL/min. The LC apparatus was coupled with a 2D linear ion trap mass spectrometer (Orbitrap Elite ETD; Thermo Fisher, Waltham, MA, USA) operated using Xcalibur 2.2 software (Thermo Fisher). A full-scan MS was performed in the Orbitrap over a range of 400 to 2000 Da and a resolution of 120,000 at *m*/*z* 400. Internal calibration was performed using the ion signal protonated dodecamethylcyclohexasiloxane ion at *m*/*z* 536.165365 as the lock mass. The 12 data-dependent MS/MS scan events were followed by one MS scan for the 12 most abundant precursor ions in the preview MS scan. The *m*/*z* values selected for MS/MS were dynamically excluded for 60 s with a relative mass window of 15 ppm. The electrospray voltage was set to 2.0 kV, and the temperature of the capillary was set to 200 °C. The MS and MS/MS automatic gain control was set to 1000 ms (full scan) and 300 ms (MS/MS), or 3 × 10^6^ ions (full scan) and 30,000 ions (MS/MS) for maximum accumulated time or ions, respectively. De Novo Peptide sequencing was carried out using PEAKS studio (version 10.5, Bioinformatics Solutions Inc., Waterloo, ON, Canada). The MS/MS spectra were analyzed using the de novo function. For peptide identification, a 10 ppm mass tolerance was permitted for intact peptide masses and 0.1 Da for HCD fragment ions, with oxidized methionine, acetyl (protein N-terminal), and deamination (asparagine) as variable modifications. The peptide–spectrum match (PSM) was then filtered based on 50% of the average local confidence (de novo score).

### 2.8. Wound Healing Assay in Animal Model

Six-week-old male Sprague Dawley (SD) rats (264.6 ± 7.0 g) were acquired from Bio LASCO Taiwan Co., Ltd. (Taipei, Taiwan), to assess the wound healing potential of perch hydrolysate. These rats were kept in standard cages with relative humidity maintained at 30–70%, temperature controlled to 22 ± 4 °C, and subjected to a 12 h light-dark cycle. They had free access to a commercial pellet diet and water ad libitum. The protocol was approved by the Institutional Animal Care and Use Committee of the Agricultural Technology Research Institute under codes 109093 and 110005. Preliminary groups of rats were studied to confirm the oral dosage of the treatment groups using varying doses of PH. A group of five SD rats were orally administered PH30 at a dose of 1972 mg/kg body weight for 21 days, with a daily single dose administered via gavage for 7 days. The control group was administered RO water.

The rats were given an intraperitoneal injection of 50 mg/kg bw Zoletil 50 for anesthesia. The fur surrounding each wound was shaved. A circular mark with a diameter of 2 cm was drawn on the back of each rat and a full-thickness wound was made with a sterilized instrument to the depth of the dermis. The wounds were then covered with 3M Tegaderm dressing. The dressings were taken off on days 1, 2, 3, 5, 7, and 21 post-surgery, and the wounds were observed and photographed using Image J (Version 1.54, National Institutes of Health, MD, USA) to measure the wound size. The percentage of wound contraction at varying days after the wound had been made, termed the wound healing rate (%), was computed by applying the following formula:

$$\mathrm{Wound}\;\mathrm{Healing}\;\mathrm{Rate}\;(\%)=\left[\frac{Would\;area\;on\;day\;0-Would\;area\;on\;day\;x}{Would\;area\;on\;day\;0}\right]\times 100\%$$

x is the corresponding time point.

### 2.9. Analytical Assays for Fibronectin, Procollagen I, and Hyaluronan in Human Fibroblasts

Human dermal fibroblast cells (890510-01F ATIT) were seeded at 5 × 10^5^ cells/well in a 6-well microplate. Once the cells had adhered, the medium was replaced with DMEM medium devoid of fetal bovine serum. Simultaneously, the sample was added, and the cells were incubated for 2 days at 37 °C in a humidified atmosphere consisting of 95% air and 5% CO_2_. The supernatant was subsequently collected and used to detect fibronectin, procollagen I, and hyaluronan levels quantitatively through an enzyme-linked immunosorbent assay (R&D systems, Minneapolis, MN, USA). We measured the absorbance value at a wavelength of 450 nm with a microplate reader, plotted it against a standard curve of known concentrations and their respective absorbance, and calculated the concentration of the sample. The negative control group (NC) was cultured in DMEM medium, the positive control group (PC) was treated with 5% fetal bovine serum, and all samples were analyzed in triplicate.

### 2.10. Statistical Analysis of Data

The statistical analysis in this study was performed using SAS software (Statistical Analysis System, SAS; Version 9.4 TSIM5, SAS Institute Inc., Cary, NC, USA). The mean ± standard error (mean ± SEM) was used to present the values of the experimental results. To identify differences between groups, the experimental data were analyzed using one-way analysis of variance (one-way ANOVA) and compared using Duncan’s multiple range test.

## 3. Results

### 3.1. Upcycled Perch Hydrolysates from Perch Side Steams

After high-pressure extraction of gutted perch, the resulting side streams of perch essence possess a sludge-like consistency and can undergo hydrolysis through the addition of enzymes without the need for any further grinding or homogenization. Table 1 presents the α-amino group amount and its degree at different times using the designed complex enzyme. The side streams of perch essence enhance the amino group amount with time of hydrolysis, thereby increasing the degree of hydrolysis. Table 1 demonstrates a higher rate of hydrolysis for up to five hours, subsequently slowing down. For subsequent analysis, the hydrolysate gained after five hours of partial hydrolysis was selected due to its operating efficiency and anticipated bioactive peptide availability. The PH of perch side streams can be achieved with a hydrolysis time of five hours, resulting in a high degree hydrolysis of 43.1% and an α-amino group concentration of 18.66 ± 0.32 mg/mL.

### 3.2. Protein Profiles and Amino Acid Composition in PH

Table 2 shows the molecular weight distribution of perch essence and PH, obtained using gel filtration chromatography. While perch essence was predominantly protein, with approximately 62.9% of the proteins having molecular weights greater than 2.3 kDa, the PH was predominantly peptides and amino acids, accounting for 81.6% of the total proteins. This finding indicates that the complex enzymes utilized in this study effectively catalyzed the hydrolysis of proteins into peptides and amino acids. Peptides with molecular weights (MWs) ranging from 190 to 2300 Dalton account for 52% of the PH, while peptides or amino acids with molecular weights below 190 Da account for 29.6%. Assuming an average molecular weight of 110 Da for an amino acid, the majority of peptides in PH are composed of between 5 and 22 amino acids.

The composition of free amino acids in PH is shown in Table 3. The five amino acids with the highest concentrations of free amino acids were as follows: leucine, lysine, valine, arginine, and isoleucine, in that order. It is noteworthy that the branched-chain amino acid content was found to be very high. This phenomenon may be attributed to the distinct hydrolysis sites of these enzymes. In addition to the highly abundant amount of leucine and valine in PH, lysine and arginine are the primary amino acids that are cleaved by the enzymes, as they are positively charged amino acid residues and typically found on the surface of proteins [24].

The hydrolyzed amino acid composition of protein in PH consists primarily of glutamic acid, aspartic acid, glycine, lysine, and alanine (Table 4). The composition of hydrolyzed amino acids differs from that of free amino acids due to the higher content of amino acids. Consequently, the hydrochloric acid-hydrolyzed amino acids from the major peptides of PH are different from those hydrolyzed by the enzymes utilized in this study. Fish proteins generally have elevated levels of essential amino acids such as lysine and leucine, as well as non-essential amino acids including aspartic acid, glutamic acid, and alanine [25], which is consistent with the findings of this study (Table 4). As demonstrated in Table 4, the hydrolyzed amino acids of PH contain 169 mg/100 g of hydroxyproline, which is an indicator of collagen. This finding indicates the presence of a significant quantity of collagen peptides in PH. The hydrolyzed amino acids found in the perch essence in [25] have a composition nearly identical to the abundance order of the perch hydrolysate content in this study. This suggests that enzyme hydrolysis could be a valuable method to recover essential amino acids that would otherwise be discarded as waste.

### 3.3. Effect of PH on Wound Healing Rate in the Animal Model

**Figure 1:**
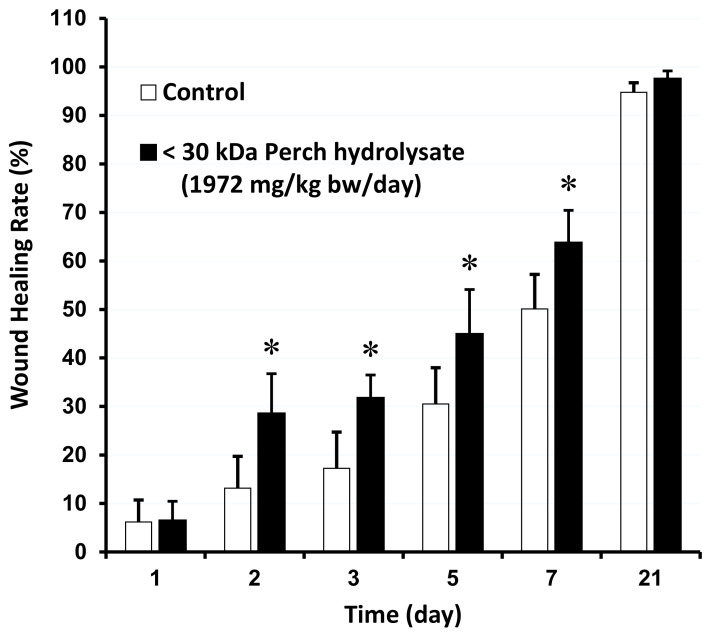
Changes in wound healing rate after oral feeding of perch hydrolysate with molecular weight less than 30 kDa in SD rats. Experiment was carried out by feeding SD rats with PH30. Data are presented as mean ± S.D., n = 5. * *p* < 0.05 (one-way ANOVA test).

Many fish peptides have been shown to promote wound healing [26,27]. However, most studies have been conducted in vitro or on the effects of topical application. To determine whether orally, the recovered peptide had any effect on wound repair, three different doses of PH were administered to SD rats. Although the oral administration of PH seemed to have a wound healing activity after three days (Figure S1), there was only a statistically significant difference at 7 days. A total of 7–29 small amino acid peptides obtained from the skin of silver carp contain significant amounts of peptides that may provide beneficial effects in patients requiring reconstructive treatment [28]. Therefore, there is an expectation that smaller proteins or peptides may have a more pronounced effect [29]. A further statistically significant increase in the rate of wound healing was observed on the second day of feeding with PH30 obtained by tangential flow filtration (Figure 1 and Figure S2).

### 3.4. Effects of PH on Fibroblasts Through Secreting Fibronectin, Procollagen I, and Hyaluronan

**Figure 2:**
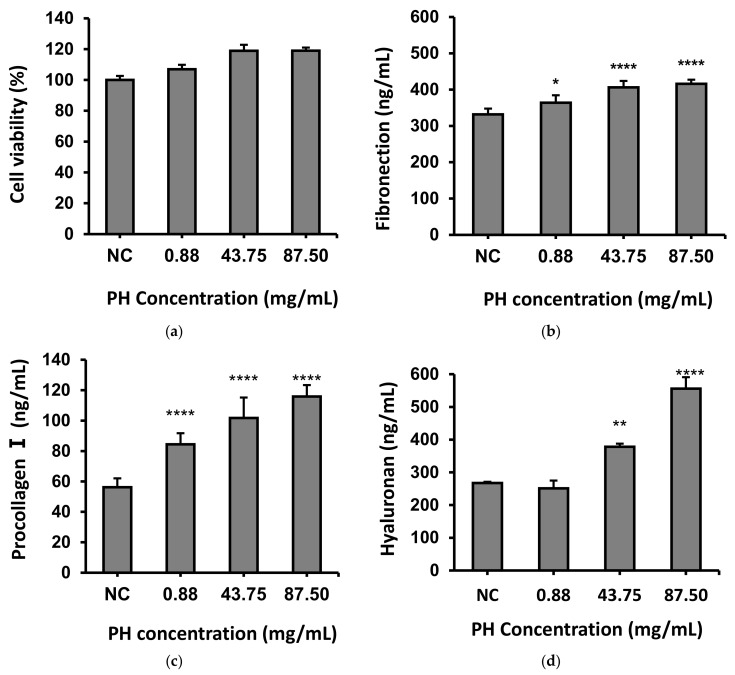
Effect of perch hydrolysates after addition to fibroblasts for 48 h. (**a**) Fibroblast viability; (**b**) fibronectin secretion; (**c**) procollagen I secretion; (**d**) hyaluronan secretion. NC means negative control, which was not added. Data are presented as mean ± S.D., n = 6. * *p* < 0.05, ** *p* < 0.01, and **** *p* < 0.0001 (one-way ANOVA test).

Spent yeast waste streams containing bioactive peptides from fermentation processes have been shown to enhance the production of metabolites, such as fibronectin, procollagen I, and hyaluronic acid [30]. Although there is no statistical difference, it appears that PH enhances fibroblast viability, as shown in Figure 2a. In addition, it can be concluded that PH is not cytotoxic to fibroblasts. As expected, the effect of PH on fibroblasts with an incubation time for 48 h significantly increased the secretion of fibronectin (Figure 2b), procollagen I (Figure 2c), and hyaluronan (Figure 2d), which are major components of the skin’s extracellular matrix (ECM).

### 3.5. Relative Abundance of Peptide Sequence in Perch Hydrolysates

Table 5 shows the amino acid sequence of the more abundant peptides in PH, almost all of which have an MW below 1200 kDa. Among them, there are relatively high abundances of FPSLVRGP and GGLLCTHYNRLAV. These two more abundant peptides in PH are characterized by more residues of electron-rich groups (e.g., aromatic groups, imidazole and pyrrole rings), more hydrophobic groups, hydrophobic amino acid residues at the N end, and groups which can easily form a hydrogen bond at the C end.

## 4. Discussion

Wound healing incurs various challenges, making it an important topic in medicine. Short-chain peptides from fish protein hydrolysates possess wound healing properties that may represent a solution. Previous research has shown that perch essence has measurable amounts of soluble protein and α-amino groups at 66.80 ± 0.56 mg/mL and 10.63 ± 0.09 mg/mL, respectively [31]. The protein analysis results indicate that residual side streams of perch essence are hydrolyzed to PH, which still contain many proteins, peptides, and amino acids. The potential benefits of the fish by-products are derived from the transformation of the biomass by means of exogenous enzyme hydrolysis, which avoids the potential risks of biological and chemical contamination [32]. From the perspective of upcycling, the resulting PH recovered about 9.22% of solids from the side streams of perch essence following enzymatic hydrolysis. Of the solids recovered, 715.7 ± 19.4 mg/g were soluble proteins. Subsequently, the PH was concentrated via ultrafiltration through a 30 kDa membrane, yielding PH30 with 887.2 ± 6.3 mg/g of soluble protein.

A wide range of bioactive properties, such as antioxidant, antihypertensive, anticoagulant, and immunomodulatory properties, have been demonstrated in peptides obtained by enzymatic hydrolysis [33,34,35,36]. Numerous studies indicate that bioactive fish peptides tend to have low molecular weights and are composed of oligopeptides containing between 2 and 10 amino acid residues with a molecular weight of less than 1.2 kDa [15,37,38]. With an average molecular weight of 110 Da per amino acid, the PH comprises 36.8% oligopeptides that contain less than 10 amino acid residues whose molecular weights range from 190 Da to 1.2 kDa, as presented in Table 2. In a systematic review and meta-analysis, supplementation with either arginine or glutamine was shown to benefit wound healing [39]. High levels of glutamic acid and arginine in the PH (Table 3 and Table 4) can contribute to the same effect. Furthermore, collagen peptides from chum salmon skin could accelerate the process of wound healing in rats [40]. Low-molecular-weight (<3 KDa) collagen peptides with in vitro wound healing activities were obtained from fish skin of the unicorn leatherjacket (*Aluterus monoceros*) [41]. The majority of peptides and amino acids in PH are shown in Table 2. The raw material is derived from the hydrolysate of by-products of perch essence production, including fish scales. Consequently, it is abundant in collagen peptides (see Table 4). The high wound repair ability of oral administration might be related to the presence of these peptides with a molecular weight of less than 2.3 kDa, except amino acids. Short sequences with potential biological activities were identified in 19 peptide sequences in prior research, including the antihypertensive sequence of the FP [42]. As shown in Table 5, our peptide sequences beginning with FP suggest that PH may also exert similar effects. Rémond et al. found that the FPSIVGRP fragment sequence had antihypertensive effects [43]. This sequence is very similar to the FPSLVRGP found in our study. These two peptides are almost identical, except for the difference between leucine and isoleucine and the difference in order between glycine and arginine. Further study is required to explore the antihypertensive effect of the FPSLVRGP sequence.

In Figure 2, the higher concentration of PH added induced more secretion in a dose-dependent manner. Signal peptides from many sources have been shown to trigger signaling pathways that stimulate fibroblasts to produce collagen, fibronectin, and laminin to improve wound healing [44]. Our signal peptides from the side stream of the perch essence are also effective in promoting the secretion of these substrates that are associated with wound repair or skin conditions. Inflammation, cell proliferation, and remodeling are recognized as the crucial stages of wound healing. The intricate process of tissue repair is driven by the proliferation of parenchymal cells induced by specific growth factors, involving mediators and hemocytes, as well as the production of the ECM [45]. The ECM performs a crucial role in regulating cellular activities, including cell growth, proliferation, and migration. It can trigger apoptosis through signaling molecules generated by the proteolysis of the ECM to form soluble peptides, which are known as matrikines [46,47]. Matrikines regulate inflammatory, fibrogenic, or reparative actions by modifying the phenotype and function of fibroblasts during wound healing [48]. The primary element of the skin’s ECM is hyaluronan, an unsulphated glycosaminoglycan that plays a role in inflammatory responses and tissue regeneration processes and is frequently applied as a wound dressing [49]. The use of topical or oral matrikine-like substances may improve the ability of wounds to heal. In our study, the PH-containing substances are similar to matrikines. These branched-chain amino acids (leucine, isoleucine, and valine) play a pivotal role in promoting protein synthesis. Also, glycine, proline, hydroxyproline, and collagen peptide in PH contribute to collagen formation. Most importantly, matrikine in PH induces the production of ECM substances such as hyaluronan.

Peptides applied orally should avoid gastrointestinal digestion to preserve their bioactivities and structures for wound repair. Peptides with C-terminal proline or hydroxyproline residues can be absorbed through the intestine intact via paracellular and transcellular routes [50]. It has been reported that peptides containing proline are typically impervious to degradation by digestive enzymes [42,51,52]. The bioactive peptide FPSIVGRP can be transported into the bloodstream by releasing mucosal peptidases, and withstand plasma peptidases, resulting in an antihypertensive impact [43]. Therefore, the C-terminal proline-containing FPSLVRGP and other peptides containing proline in our PH may have the ability to pass through the intestines and display wound healing properties. The results of this study suggest that orally administered PH, which is rich in signal peptides, can cross the intestinal mucosa and be delivered to wounds via blood vessels to promote wound healing through the secretion of fibronectin, procollagen I, and hyaluronan by the ECM through the transduction of the signal.

PH could be applied for not only topical purposes but also dietary supplements to speed up the wound healing process by providing amino acids and non-digestible matrikine-like peptides. The present study focuses on oral ingestion as opposed to topical application. With respect to the reduction in waste, the utilization of PH from the hydrolyzed side streams of perch essence to promote wound healing is of significance in the ESG context.

## 5. Conclusions

Through a designed commercial complex enzyme via a proprietary pressure extraction technique, we found that perch hydrolysates upcycled from perch side streams contain various beneficial peptides and amino acids. Short-chain peptides are necessary for wound repair and matrikine prompt fibroblasts to generate fibronectin, procollagen I, and hyaluronan. Our upcycling technique is effective in recovering the proteins left over after perch extraction in the production of perch essence. In light of this, our perch hydrolysates could be developed as dietary nutraceuticals or functional foods to promote wound healing, particularly in undernourished subjects, post-surgical patients, or post-partum women.

## Figures and Tables

**Table 1:** α-Amino group concentration and hydrolysis degree during enzymatic hydrolysis at different times.

Hydrolysis Time (h)	α-Amino Group Concentration (mg/mL)	Hydrolysis Degree (%)
0	4.62 ± 0.34	10.68 ± 0.83
2	9.63 ± 0.28	22.28 ± 0.66
4	12.43 ± 0.17	28.75 ± 0.40
5	18.66 ± 0.32	43.14 ± 0.75
6	18.88 ± 0.64	43.67 ± 1.49
10	21.49 ± 0.09	49.69 ± 0.21
24	24.84 ± 0.23	57.45 ± 0.53

Note: Table shows α-amino group concentration and hydrolysis degree in perch hydrolysates during enzymatic hydrolysis. Data are mean ± SD from four experiments (n = 4), showing increasing trends indicative of protein hydrolysis.

**Table 2:** Analysis of molecular weight distribution in perch essence and perch hydrolysates using gel filtration chromatography.

Molecular Weight (Da)	Percentage (%)	
	Perch Essence	Perch Hydrolysate (PH)
>2300	62.9	18.4
2300–1200	14.5	15.2
1199–580	10.7	15.8
579–240	7.9	17.0
239–189	1.3	4.0
<189	2.7	29.6
Average	5843	1289

Note: Table displays molecular weight distribution in perch essence and hydrolysates (PH) analyzed using gel filtration chromatography, highlighting protein breakdown during hydrolysis.

**Table 3:** Concentrations of free amino acids in perch hydrolysates.

Amino Acid	Concentration (mg/100 g)
Leucine	212.84
Lysine	113.29
Valine	96.22
Arginine	94.60
Isoleucine	87.41
Phenylalanine	84.75
Methionine	62.24
Tyrosine	50.58
Proline	20.02
Carnosine	8.80
Tryptophan	4.80
β-Aminoisobutyric Acid	4.75
DL-plus allo-δ-Hydroxylysine	4.52
Cystathionine	4.47
Cysteine	3.44
Ornithine	2.31
γ-Aminobutyric Acid	2.32
Hydroxyproline	N.D.
Total	890.58

Note: Free amino acid concentrations in perch hydrolysates demonstrate that leucine is the most abundant. ‘N.D.’ stands for ‘Not Detected’.

**Table 4:** Concentration of hydrolyzed amino acids in perch hydrolysate.

Hydrolyzed Amino Acid Profiles	Concentration (mg/100 g)
Glutamic acid	957
Aspartic acid	660
Glycine	631
Lysine	574
Alanine	500
Leucine	499
Arginine	443
Proline	383
Threonine	332
Valine	332
Isoleucine	290
Serine	283
Phenylalanine	256
Methionine	202
Hydroxyproline	169
Histidine	166
Total	6677

Note: Table shows concentrations of hydrolyzed amino acids in perch hydrolysates, highlighting high levels of glutamic acid, aspartic acid, and glycine.

**Table 5:** Relative abundance of peptide sequence in perch hydrolysates.

Sequence	Relative Abundance (%)
FPSLVRGP	6.21%
GGLLCTHYNRLAV	5.16%
GALGMLGDYSLV	2.89%
LNLAMNALDLYL	2.87%
MGLLCKGSPATP	2.79%
TSEGTRVAPW	2.66%
QLGMLMYGPGLTGQ	1.99%
PEDVLLDAFKVLDPKYHRT	1.90%
MGLGTPWLNQF	1.72%
GMTGLWPW	1.49%

Note: The table shows FPSLVRGP being the most prevalent in the relative abundance of peptide sequences in perch hydrolysates.

## Data Availability

Data are contained within the article and Supplementary Materials.

## Supplementary Materials

The following supporting information can be downloaded at: https://www.mdpi.com/article/10.3390/cimb47010057/s1.
