# Supplementary material for: Perch Hydrolysates from Upcycling of Perch Side Streams Accelerate Wound Healing by Enhancing Fibroblasts to Secrete Procollagen I, Fibronectin, and Hyaluronan
Source: Curr Issues Mol Biol. 2025 Jan 16;47(1):57. doi: 10.3390/cimb47010057 (PMC11763970; doi:10.3390/cimb47010057)
Supplement: Supplementary file 1 [file cimb-47-00057-s001.zip › cimb-3399551-supplementary.pdf]

(**A**)

(**B**)

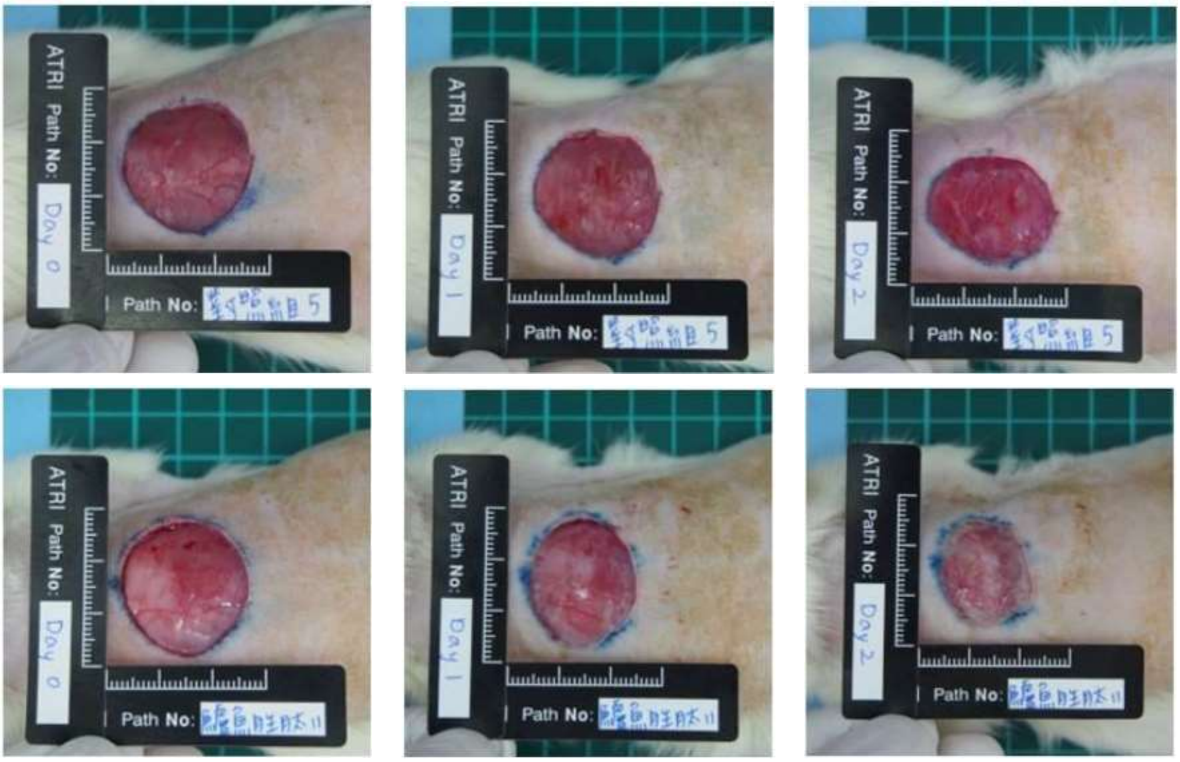

Day 0 **Day 1** **Day 2**

Figure S1. Wound photographs of SD rats on the first to second day. (**A**) control, (**B**) PH30.

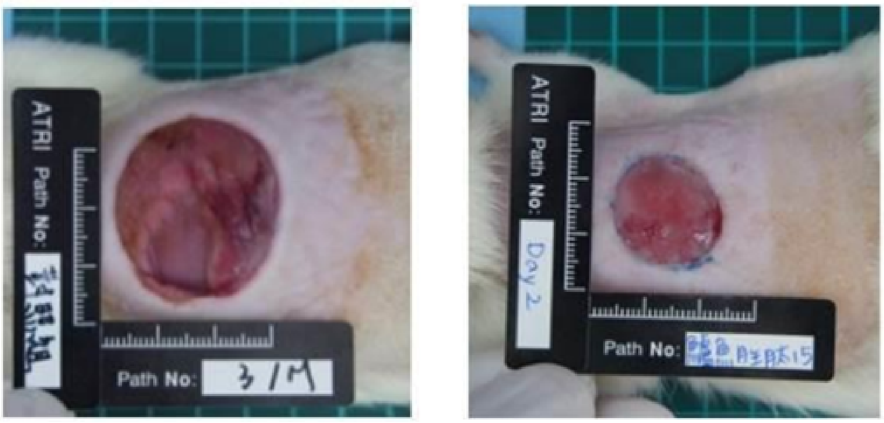

Control **<30 kDa Perch hydrolysate**

Figure S2. Photograph of the wound (Day 2).
